# Supplementary material for: Impact of COVID-19 on Antimicrobial Consumption and Spread of Multidrug-Resistance in Bacterial Infections
Source: Antibiotics (Basel). 2022 Apr 18;11(4):535. doi: 10.3390/antibiotics11040535 (PMC9025690; doi:10.3390/antibiotics11040535)
Supplement: Supplementary file 1 [file antibiotics-11-00535-s001.zip › antibiotics-1674293-supplementary.pdf]

Table S1. Prevalence of vancomycin-resistant *Enterococcus* isolates from clinical and surveillance samples.

VRE species	Ward				ICU			
	March 2018 - September 2019	March 2020 - September 2021	% change	*p*	March 2018 - September 2019	March 2020 - September 2021	% change	*p*
**Clinical**								
E. faecium	0.46	0.68	+50.1	**< 0.001**	1.48	1.90	+28.6	0.094
E. faecalis	0.01	0.01	+19.4	0.439	0.04	0.02	-37.5	1.000
**Surveillance**								
E. faecium	0.45	0.42	-8.2	0.424	3.75	1.31	-65.1	**< 0.001**
E. faecalis	0.01	0.01	+39.3	0.395	0.04	0.04	+24.9	0.731

Values are presented as infection cases/1000 patient-days
VRE, vancomycin-resistant *Enterococcus;* ICU, intensive care unit.

Table S2. Prevalence of carbapenem-resistant *Enterobacteriaceae* isolates from clinical and surveillance samples.

CRE species	Ward				ICU			
	March 2018 - September 2019	March 2020 - September 2021	% change	*P*	March 2018 - September 2019	March 2020 - September 2021	% change	*P*
**Clinical**								
K.pneumoniae	0.15	0.22	+45.4	**< 0.001**	0.75	1.25	+67.6	**< 0.001**
Escherichia coli	0.06	0.09	+58.0	**< 0.001**	0.09	0.13	+36.1	0.281
Enterobacter spp.	0.04	0.03	-36.8	0.175	0.14	0.08	-42.7	0.204
Serratia spp.	0.01	0.01	+49.2	0.251	0.07	0.08	+14.5	0.611
Klebsiella spp.	0.01	0.01	-12.5	0.991	0.05	0.07	+33.8	0.451
Citrobacter spp.	0.01	0.01	+118.8	0.06	0.02	0.03	+38.8	0.713
Others	0.00	0.00	-	0.143	0.00	0.01	+	0.219
**Surveillance**								
K. pneumoniae	0.29	0.44	+52.4	**< 0.001**	2.58	2.86	+10.7	**0.009**
Escherichia coli	0.06	0.09	+45.2	**0.001**	0.58	0.48	-17.5	0.521
Enterobacter spp.	0.04	0.06	+23.9	0.066	0.70	0.44	-38.0	**0.016**
Serratia spp.	0.00	0.00	+19.4	1	0.06	0.04	-21.9	0.764
Klebsiella spp.	0.02	0.03	+85.7	**0.010**	0.31	0.26	-15.3	0.726
Citrobacter spp.	0.02	0.02	+30.7	0.176	0.18	0.11	-37.5	0.235
Others	0.00	0.00	+258.1	0.327	0.01	0.03	+108.2	0.428

Values are presented as infection cases/1000 patient-days
CRE, carbapenem-resistant *Enterobacteriaceae;* ICU, intensive care unit; *Enterobacter* spp.: *E. cloacae, E. cancerogenus, E. hormaechei, E. kobei; Serratia* spp.: *S. liquefaciens, S. marcescens; Klebsiella* spp.: *K. aerogenes, K. oxytoca, K. ozaenae, K. variicola; Citrobacter* spp.: *C. amalonaticus, C. braakii, C. koseri, C. freundii*; Others: *Cronobacter sakazakii* complex, *Hafnia alvei, Kluyvera cryocrescens, Providencia rettgeri, Providencia stuartii,* and *Raoultella planticola.*
